# Factors Predicting the Ability to Perform Activities of Daily Living among Stroke Patients in Rural Community Southern Thailand

**DOI:** 10.21315/mjms2024.31.5.18

**Published:** 2024-10-08

**Authors:** Nipaporn Kuakool, Dusanee Suwankhong, Tum Boonrod, Chamnan Chinnasee

**Affiliations:** 1 Department of Public Health, Thaksin University, Phatthalung, Thailand; 2 Department of Health and Sport Science, Faculty of Education, Mahasarakham University, Thailand

**Keywords:** stroke, activities of daily living (ADL), rehabilitation, rural health services

## Abstract

**Background:**

The aim of this study was to explore the factors associated with the ability to perform activities of daily living (ADL) among post-stroke patients participating in outpatient physical rehabilitation programmes in community hospital in southern Thailand.

**Methods:**

In this cross-sectional study, data were collected from 258 patients diagnosed with stroke through the HOSxP programme from 2018 to 2022. Patients’ ADL were assessed using the Barthel Index measurement. Characteristics were described by percentages and medians (IOR). Associations of categorical variables were examined using the chi-squared test, and multiple logistic regressions were employed to identify factors predicting Barthel Index scores among stroke patients. Patients’ capacity levels were categorised based on Barthel Index scores and rehabilitation criteria, and unadjusted and adjusted odds ratios (OR/AOR) were presented, with a significance level of 0.05.

**Results:**

Of the 258 stroke patients, 59.30% were male, with a median age of 67 years old. Significant differences in Barthel Index scores were found with respect to gender, age, marital status, comorbidities and duration of rehabilitation (*P* < 0.05). Binary logistic regression analysis indicated that male stroke patients (AOR = 1.89; 95% confidence interval [CI]: 1.10, 3.26), individuals with single marital status (AOR = 4.62; 95% CI: 1.58, 13.49), absence of comorbidities (AOR = 0.53; 95% CI: 0.29, 0.98), and rehabilitation duration of less than five days (AOR = 4.38; 95% CI: 2.14, 8.96) were more likely to achieve independence in ADL with higher Barthel Index scores (*P* < 0.05).

**Conclusion:**

Several factors contribute to the effective planning of care and rehabilitation for stroke patients in rural area. Stroke rehabilitation programmes in this region should consider various elements, including patient characteristics, family involvement and clinical factors, to improve ADL performance.

## Introduction

A stroke is a sudden disruption of blood flow in the blood vessels of the brain and can affect a specific region or the entire brain. It can lead to cerebral haemorrhage, brain vessel rupture or cerebral infarction. Ischaemic stroke is the most common type, accounting for approximately 80% of all cases (1). Stroke is also the second leading cause of death and disability worldwide (2). In 2017, Thailand recorded 81,325 new cases of stroke among individuals aged 15 years old and above, resulting in an incidence rate of 151.59 per 100,000 people. Therefore, stroke is considered a critical health problem in Thailand (3).

Stroke often results in physical impairments, emotional distress and an increased burden on caregivers, which can hinder the rehabilitation progress, increase the risk of disability and reduce overall quality of life. Comprehensive stroke rehabilitation programmes play a vital role in supporting stroke patients in their recovery, restoring functionality, and improving their health and well-being. The main goals of stroke rehabilitation focus on improving daily activities and motor skills (4).

The stroke rehabilitation guidelines introduced by the Ministry of Public Health of Thailand (5) recommend the use of the Barthel Index, a 20-point scale, to assess stroke patients’ ability to perform activities of daily living (ADL). This assessment tool evaluates 10 items of daily living: eating, bathing, dressing, bowel and urinary control, transferring between beds, using the toilet, and ascending and descending stairs. Based on their Barthel Index scores, stroke patients were divided into two categories that indicate their ability to perform daily tasks: i) an intermediate care group, consisting of patients with scores below 15 or those experiencing complications, and ii) a community discharge group, comprising patients with scores of 15 or higher.

The Barthel classification system plays a vital role in tailoring rehabilitation programmes to individual patient abilities and post-stroke challenges, thereby improving overall functional performance and reducing disability. However, many factors, such as personal traits (age and gender); family circumstances (availability of supportive caregivers and marital status); and clinical conditions (muscle weakness, post-stroke complications, pneumonia, urinary tract infections and pressure sores), can impact stroke patients’ capacity to carry out ADL (6). Additional characteristics, such as stroke severity, recurrent episodes, advanced age, and limited financial and nutritional resources, have been associated with a decreased ability to perform ADL following a stroke. Moreover, depression, fatigue, and inadequate environmental support further impede stroke patients’ rehabilitation progress (7–14).

The healthcare system should aim to facilitate stroke patients’ complete independence in ADL through comprehensive interventions. Patients should undergo holistic rehabilitation during their hospital stays and continue to receive care after returning home (12). Healthcare professionals in community hospitals near patients’ residences should prioritise post-crisis care, including rehabilitation treatments during hospitalisation and planning rehabilitation programmes for ongoing recovery at home after discharge (6, 15, 16).

Addressing the healthcare needs of stroke patients in Thailand poses significant challenges, particularly in rural areas, where resources are relatively limited and infrastructure is somewhat underdeveloped (17). From 2018 to 2022, Cha-Uat Community Hospital provided physical therapy to over 1,000 stroke patients. According to the guidelines set by the Ministry of Public Health of Thailand, patients must undergo assessments using the Barthel Index before being discharged. It indicated that a significant proportion, ranging from 53.85% to 77.78%, scored between 0 and15 on the Barthel Index, demonstrating an inability to independently perform daily activities. Approximately 20% of these patients experience complications such as muscle strain, shoulder dislocation, and pressure sores (18). Thus, there is an urgent need for sustainable rehabilitation programmes aimed at enhancing patients’ ability to live independently.

Numerous factors have been identified as influencing stroke patients’ ability to perform ADL in other societies (19–21). However, there is insufficient data on these factors, especially in community hospital in southern Thailand. Therefore, this study aimed to fill this knowledge gap to guide the development of effective long-term treatment plans and personalised healthcare rehabilitation programmes for stroke patients in rural regions.

## Methods

### Study Design and Participants

This study extracted data from stroke patients enrolled in the HOSxP programme from 2018 to 2022, totalling 1,287 individuals. The HOSxP dataset comprises personal information, demographic details and medical treatment records extracted from National Health Insurance (NHI) claims filed by medical institutions with the National Health Insurance Service (NHIS). These records encompass both inpatient and outpatient visits for each patient. The study sample included individuals who met the following criteria: i) aged 18 years old or older; ii) diagnosed with a first acute stroke by a neurologist; iii) received outpatient physical therapy treatment between 1 January 2018 and 31 December 2022; iv) unable to independently perform ADL; v) a resident of Cha-Uat District and vi) conscious and able to provide clear answers to questions. Patients with mental illness, aphasia, cognitive impairment or who declined to participate were excluded from the analysis. Purposive sampling was used by physical therapists with over 5 years of experience to select stroke patients referred to the physical therapy department of Cha-Uat Community Hospital. The sample size was determined using Cochran’s formula (22):


$$n=\frac{NZ_{\propto /2}^{2}P(1-P)}{e^{2}(N-1)+Z_{\propto /2}^{2}P(1-P)}$$

Thus, the minimum required sample size for this study was 258 cases (*Z* = 1.96 at 0.05 significance level, *P* = proportion of 30%, *e* = precision at 5).

### Data Collection Process

After obtaining ethical approval from the Thaksin University Ethics Committee on Human Research, we informed the committees of community hospital about background of study and asked permission to access the HOSxP programme for data collection. Later, we collected patient medical history data from this programme. This software application, developed by the Ministry of Public Health of Thailand (5), is specifically designed to store patient information. To ensure confidentiality, individual patient identities were excluded from the dataset.

### Data Sources/Measurement

The sociodemographic characteristics of the study sample included gender, age and marital status. Medical data included information on comorbid conditions, such as hypertension, diabetes and hyperlipidaemia, as well as details on stroke type, affected side, blood pressure values (systolic and diastolic), duration of diagnostics before the initiation of rehabilitation and number of rehabilitation days.

### Barthel Index for Activities of Daily Living Assessment Tool

The Barthel Index is used as an assessment tool to assess the ability of stroke patients to carry out ADL (18). The scale assigns a total score ranging from 0 (indicating the poorest performance in ADL) to 20 (reflecting optimal performance). The 10-item index has been validated for clinical use in stroke rehabilitation and includes rating combinations of: i) 0 and 1; ii) 0, 1 and 2 or iii) 0, 1, 2 and 3. Prior to discharge, all patients were assessed using the Barthel Index tool to determine their level of performance in ADL. Stroke patients were classified into two groups based on their Barthel Index scores, which indicate their ability to perform ADL: i) an intermediate care group, comprising patients with scores below 15 or those with complications, and ii) a community discharge group, with scores of 15 or higher.

### Statistical Analysis

Data analysis was carried out using STATA version 12.0. Descriptive statistical analysis was used to summarise the distribution of data, including the calculation of means, standard deviations, percentages and frequencies. A chi-square test of independence was used to assess the relationship between the categorical variables. Multiple logistic regression analysis was conducted to identify factors predicting Barthel Index scores among stroke patients undergoing care at the physiotherapy department of Cha-Uat Community Hospital. In addition, patients were categorised into two subgroups based on their Barthel Index scores and rehabilitation criteria. Both unadjusted odds ratio (OR) and adjusted odds ratio (AOR) were reported, with statistical significance set at 0.05.

## Results

### Sociodemographic and Clinical Characteristics

A total of 258 participants were included in the study, with a median age of 67 years old and approximately 59% male. The majority of participants were married (90.31%) and had comorbid conditions (75.58%), primarily ischaemic stroke (75.58%). Roughly half of the participants experienced a stroke on the right side (56.20%). Nearly 70% exhibited high systolic blood pressure (≥ 140 mmHg), while approximately 35% had elevated diastolic blood pressure (≥ 90 mmHg). Furthermore, 64.34% of patients received therapy within 14 days of diagnosis and 80.62% underwent rehabilitation for less than 5 days (Table 1).

The data also indicated that 51.96% of stroke patients were male and had a Barthel Index score of less than 15, while 64.10% had a score of 15 and above. The majority of patients in both groups were approximately 60 years old and married. Patients with Barthel Index scores below 15 reported a higher prevalence of comorbid diseases than those with scores above 15. Ischaemic stroke was the most common diagnosis in both groups. About 66% of individuals with Barthel Index scores below 15 had systolic blood pressure readings above 140 mmHg. Among stroke patients with Barthel Index scores above 15, around 67% had diastolic blood pressure levels exceeding 90 mmHg.

Regarding the timing of rehabilitation, among those with a Barthel Index score of less than 15, 68.63% were diagnosed less than 14 days before starting a rehabilitation programme, with a rehabilitation duration of less than 5 days. Among those with a Barthel Index score greater than 15, 61.54% had less than 14 days between diagnosis and the start of rehabilitation and 88.46% received rehabilitation for less than five days (Table 2).

### Predictors of Barthel Index Scores

Binary logistic regression was performed to assess the predictive value of various variables for Barthel Index scores among stroke patients. In this study, men were 1.89 times more likely than women to have better Barthel Index scores (AOR = 1.89; 95% CI = 1.10, 3.26). Stroke patients with a single marital status had better Barthel Index scores compared to other marital statuses (AOR = 4.62; 95% CI = 1.58, 13.49). There was a significant difference in Barthel Index scores among patients with comorbidities in the final multivariate logistic regression analysis. Stroke patients with comorbidities were 47% less likely to achieve a Barthel Index score ≥ 15 compared to those without comorbidities (AOR = 0.53; 95% CI = 0.28, 0.98). Moreover, stroke patients who received less than 5 rehabilitation days in the outpatient physical therapy department were significantly more likely to achieve a Barthel Index score of ≥ 15 compared to patients undergoing more than 5 days of rehabilitation. Specifically, the AOR for this association was 4.38, with a 95% CI ranging from 2.14 to 8.96 (Table 3).

## Discussion

This study explored the factors associating with the functional capacity of stroke patients in performing ADL. It revealed a notable association between several factors examined, including gender, marital status, comorbidities, duration of rehabilitation and the Barthel Index score, which serves as an indicator of the patient’s level of independence in ADL.

This study found that males achieved higher Barthel Index scores compared to females, accounting for 64.10% of the total participants, as shown in Table 2. Males exhibited a statistically significant likelihood of achieving better Barthel Index scores than females. This finding is consistent with a previous study (21) that highlighted males’ greater tendency to initiate neurorehabilitation within 30 days of diagnosis compared to females. While both male and female participants demonstrated improvement in Barthel Index scores, pain levels and performance on walking tests during neurorehabilitation, female stroke patients presented a poorer clinical profile upon admission than male patients. This included lower levels of independence, lower Barthel Index scores, higher pain scores and poorer performance on the 2-min walk test (2’WT). A study by Demel et al. (23) indicated that females, both in rural and urban areas, had a lower attendance rate at stroke rehabilitation sessions compared to males.

Previous research has highlighted the important role of gender in stroke recovery, with females generally recovering more slowly than males. This discrepancy is often due to the older age of female stroke patients, many of whom have experienced menopause. During this transitional phase, declining testosterone levels may hinder the ability to build muscle mass, making the recovery process more difficult (21). Furthermore, older patients who are immobile may experience muscle atrophy, which presents an additional challenge for women in regaining lost muscle mass during the recuperative phase (23). In addition, women typically exhibit lower levels of muscle activity compared to men, which contributes to reduced mobility and impedes the body’s restorative mechanisms (24). Thus, the effectiveness of rehabilitation tends to be lower in women.

In addition, females have a higher risk of complications and frailty compared to males (69% versus 59%) (*P* = 0.03) (7). The literature suggests that females have significantly higher levels of disability both before and during the acute phase of stroke, particularly in activities such as dressing, self-care and transferring from bed to chair (59% versus 37%; 57% versus 34%; 59% versus 35%, respectively) (*P* < 0.01). Following stroke onset, women face a heightened risk of disability, even after 3 months–6 months, with a greater likelihood of living alone post-stroke. This translates to a 3.5-fold increase in the probability of prolonged hospitalisation for recovery (*P* < 0.01). Therefore, gender is a critical factor influencing the level of ADL in stroke patients.

This study also found that marital status emerged as a significant factor influencing the level of ADL in stroke patients. Unmarried individuals were 4.26 times more likely to have better ADL scores than those in other marital statuses. This finding contradicts previous research suggesting that patients with family support are more likely to seek stroke treatment than single individuals (25). Marriage and support from a marital partner may promote a more favourable recovery trajectory by providing a stable and supportive environment, encouraging participation in rehabilitation activities, and promoting overall health and well-being (26). Having a supportive marital partner can also help alleviate depressive symptoms, which is crucial for post-stroke functional independence. However, mortality rates from strokes were found to be lower among unmarried individuals (26). Marital status is just one aspect that influences a patient’s ability to engage in daily activities and may be related to several other familial factors.

Comorbidities, particularly diabetes, hypertension and dyslipidaemia, are prevalent among stroke patients and significantly affect Barthel Index scores. Stroke patients with comorbidities were 47% less likely to have a Barthel Index score greater than 15 compared to stroke patients without comorbidities (AOR = 0.53; 95% CI = 0.28, 0.98). This finding is consistent with research conducted by Boehme et al. (11), who found that chronic diseases, such as diabetes, high blood pressure and dyslipidaemia, increased the risk of stroke by up to 90% because they alter blood vessel structure and function. Reduced blood flow to the brain may also reduce the autonomic control of cerebral blood vessels. In addition, the increase in neurological damage following ischaemic or inflammatory conditions of cerebrovascular disease can further disrupt the blood supply to the brain (12). Ashraf et al. (13) suggested that comorbidities may increase the risk of stroke but not necessarily affect the severity of the disease. A lack of awareness of stroke symptoms among individuals can lead to misinterpretation of symptoms and delays in treatment.

Stroke patients with conditions such as diabetes, hypertension and congestive heart failure (CHF) tend to have poorer functional outcomes (27). Specifically, patients with CHF were observed to be older, have a lower baseline functional status and were more likely to be transferred to residential care facilities compared to those without CHF. However, no significant association was detected between functional ability and comorbidities such as diabetes and hypertension (27). A Swedish study found a high prevalence of comorbidity among first-ever ischaemic stroke patients. Comorbidity was shown to double the proportion of poor outcomes at 12 months and 5 years after stroke, confirming its long-term negative influence on mortality and functional outcomes. Nevertheless, the effect of long-term rehabilitation on the functioning of stroke patients with comorbidities remains uncertain. It is important to note that the study’s results may not be generalisable due to the limited sample size and retrospective data recording accuracy (28).

According to this study, the duration of rehabilitation showed a significant correlation with Barthel Index scores in stroke patients. The findings indicate that stroke patients attending outpatient physical therapy for less than 5 days had a higher chance of achieving a Barthel Index score of 15 or higher compared to those who attended for more than 5 days, with an OR of 4.38 (95% CI = 2.14, 8.96). Moreover, previous research (29) suggests that stroke patients with low Barthel Index scores upon discharge require intensive rehabilitation provided by a multidisciplinary team. Several studies (26, 29, 30) have confirmed that patients who undergo 10 h–15 h of physical therapy rehabilitation per week demonstrate statistically significant improvements in their ability to perform ADL. Patients receiving physical therapy within this time frame are 6.5 times more likely to enhance their ability to perform daily activities compared to those receiving less than 5 h of physical therapy per week. Furthermore, it has been found that the number of weekly physical therapy hours directly affects the ability to perform daily activities, as assessed by Barthel Index scores. Increasing the intensity of physical therapy from 5 h–10 h per week positively impacts the functioning of the motor-nervous system, thereby enhancing individuals’ ability to use their arms and legs effectively (29). In general, increasing the duration of physical therapy sessions positively influences stroke patients’ ability to perform ADL. It is worth noting that the present study was conducted in a community hospital with limited health providers and it was observed that patients who received care from the physical therapy department for a shorter duration tended to have better ADL scores, thus requiring fewer follow-up appointments.

The findings of this study underscore the importance of continuing participation in physical therapy rehabilitation programmes for stroke patients to improve their ability to perform ADL. This is crucial for older patients, highlighting the importance of prioritising rehabilitation and physical therapy interventions in this population.

Several limitations of the study should be mentioned. First, it did not explore stroke patients’ long-term abilities to perform ADL, which could provide valuable insights into their rehabilitation progress over time. Moreover, the data were limited to a rural community hospital in southern Thailand, which may limit the generalisability of the findings to other regions or healthcare settings. Future research should aim to investigate additional factors that may influence the ADL of stroke patients, thereby providing a more comprehensive understanding of their rehabilitation needs. Such factors may include disease severity, duration of hospitalisation, mental health status, caregiver readiness and characteristics of the home environment. Addressing these factors may contribute to the development of more effective rehabilitation strategies tailored to the diverse needs of stroke patients.

## Conclusion

The primary aim of this study was to determine the factors associated with the ability to perform ADL among stroke patients in rural community hospital in southern Thailand. The study revealed that the ability to engage in ADL is influenced by various factors, including gender, marital status, comorbidity and duration of rehabilitation. These findings emphasise the importance of adopting a holistic approach to stroke care in rural areas that considers these factors as integral components. Therefore, healthcare providers and allied professionals in rural healthcare service areas should develop tailored rehabilitation programmes that address the specific needs of their patients. Such initiatives have the potential to mitigate disability and alleviate the burden on caregivers.

## Figures and Tables

**Table 1:** Sociodemographic and clinical characteristics of the stroke patients (*N* = 258)

Variables	Total (*N* = 258) *n* (%) a	Median (IQR)
Gender		
Male	153 (59.30)	
Female	105 (40.70)	
Age (years old)		67 (17)
< 60	79 (30.62)	
≥ 60	179 (69.38)	
Marital status		
Single	25 (9.69)	
Other	233 (90.31)	
Comorbidities		
No	63 (24.42)	
Yes	195 (75.58)	
Stroke subtype		
Non-ischaemic	63 (24.42)	
Ischaemic	195 (75.58)	
Effected side		
Right	145 (56.20)	
Left	113 (43.80)	
Systolic blood pressure (mmHg)		155 (41)
< 140	85 (32.95)	
≥ 140	173 (67.05)	
Diastolic blood pressure (mmHg)		85 (24)
< 90	168 (65.12)	
≥ 90	90 (34.88)	
Pre-physical therapy period (days)		8 (19)
< 14	166 (64.34)	
≥ 14	92 (35.66)	
Rehabilitation duration (days)		1 (2)
< 5	208 (80.62)	
≥ 5	50 (19.38)	

Note: Data were expressed as *n* (%) unless otherwise indicated; IQR = Interquartile;

a Column % presented

**Table 2:** Association between selected variables and Barthel Index score (*N* = 258)

Variables	Total (*N* = 258)	Barthel Index (BI) score		*X^2^* statistics (df = 1)	*P*-value^a^

		BI < 15 *n* = 102 (39.53)	BI ≥ 15 *n* = 156 (60.47)		

	*n* (%)	*n* (%)	*n* (%)		
Gender					
Male	153 (59.30)	53 (51.96)	100 (64.10)	3.767	0.052
Female	105 (40.70)	49 (48.04)	56 (35.90)		
Age (years old)					
< 60	79 (30.62)	28 (27.45)	51 (32.69)	0.797	0.372
≥ 60	179 (69.38)	74 (72.55)	105 (67.31)		
Status					
Single	25 (9.69)	6 (5.88)	19 (12.18)	2.794	0.095
Other	233 (90.31)	96 (94.12)	137 (87.82)		
Comorbidity					
No	63 (24.42)	32 (31.37)	31 (19.87)	4.420	0.036^a^
Yes	195 (75.58)	70 (68.63)	125 (80.13)		
Diagnostics					
Non-ischaemic	63 (24.42)	27 (26.47)	36 (23.08)	0.3849	0.535
Ischaemic	195 (75.58)	75 (73.53)	120 (76.92)		
Effected side					
Right	145 (56.20)	52 (50.98)	93 (59.63)	1.868	0.172
Left	113 (43.80)	50 (49.02)	63 (40.38)		
SBP (mmHg)					
< 140	85 (32.95)	35 (34.31)	50 (32.05)	0.142	0.705
≥ 140	173 (67.05)	67 (65.69)	106 (67.95)		
DBP (mmHg)					
< 90	168 (65.12)	64 (62.75)	104 (66.67)	0.417	0.518
≥ 90	90 (34.88)	38 (37.25)	52 (33.33)		
Pre-physical therapy period (days)					
< 14	166 (64.34)	70 (68.63)	96 (61.54)	1.350	0.245
≥ 14	92 (35.66)	32 (31.37)	60 (38.46)		
Rehabilitation duration (days)					
< 5	208 (80.62)	70 (68.63)	138 (88.46)	15.528	< 0.001^a^
≥ 5	50 (19.38)	32 (31.37)	18 (11.54)		

Note: Significant association was measured by chi-squared statistical test;

a Significant at *P* < 0.05

**Table 3:** Factors associated with the Barthel Index scores among stroke patients of the study (*N* = 258)

Variables	Unadjusted model			Adjusted model		
	
	OR	95% CI	*P*-value	OR	95% CI	*P*-value
Gender						
Female	Reference	(0.99, 2.74)	0.053	1.89	(1.10, 3.26)	0.021^a^
Male	1.65					
Age (years old)						
< 60	Reference	(0.45, 1.34)	0.372	0.63	(0.33, 1.17)	0.146
≥ 60	0.78					
Marital status						
Other	Reference	(0.85, 5.76)	0.102	4.62	(1.58, 13.49)	0.005^a^
Single	2.21					
Comorbidities						
Yes	Reference	(0.30, 0.96)	0.037	0.53	(0.28, 0.98)	0.043^a^
No	0.54					
Diagnostics						
Non-Ischemic	Reference	(0.67, 2.13)	0.535	1.26	(0.65, 2.44)	0.479
Ischemic	1.20					
Effected side						
Right	Reference	(0.42, 1.16)	0.172	0.67	(0.39, 1.15)	0.147
Left	0.70					
Systolic blood pressure (mmHg)						
< 140	Reference	(0.65, 1.88)	0.705	1.14	(0.58, 2.21)	0.696
≥ 140	1.10					
Diastolic blood pressure (mmHg)						
< 90	Reference	(0.49, 1.41)	0.518	0.85	(0.47, 1.51)	0.586
≥ 90	0.84					
Pre-physical therapy period (days)						
< 14	Reference	(0.80, 2.31)	0.246	1.50	(0.83, 2.72)	0.175
≥ 14	1.36					
Rehabilitation duration (days)						
≥ 5	Reference	(1.83, 6.68)	< 0.001	4.38	(2.14, 8.96)	< 0.001^a^
< 5	3.50					

Notes: Estimates of odds ratio from a binary logistic regression adjusted;

a Statistically significant at *P* < 0.05;

OR = odds ratio; CI = confidence interval
